# Improving efficiency of sow productivity: nutrition and health

**DOI:** 10.1186/2049-1891-4-26

**Published:** 2013-07-26

**Authors:** Sung Woo Kim, Alexandra C Weaver, Yan Bin Shen, Yan Zhao

**Affiliations:** 1 Department of Animal Science, North Carolina State University, North Carolina, Raleigh 27695, USA

**Keywords:** Colostrum, Gestation, Lactation, Milk, Nutrition, Pig

## Abstract

This reviews research focused to understand the nutrient requirement and balance to meet the needs of fetal growth, mammary growth, and milk production. This summary will handle how feeding strategies can be adjusted according to the nutrient needs for a sow to enhance productivity and health. Most research data used in this summary are based on the studies conducted by the authors between 1996 and 2013. Nutrient requirements of sows are affected by stage of gestation and parity of sows. Dietary antioxidant concentrations need to be re-evaluated for its sufficiency in sow diets especially to prevent excessive oxidative stress during late gestation and lactation. When feeding sows, consideration of phase feeding of gestating sows and parity feeding of lactating sows could enhances production longevity and health of sows. Use of selected nutrients and additives seems to help productivity and health of sows.

## Introduction

**Figure 1:**
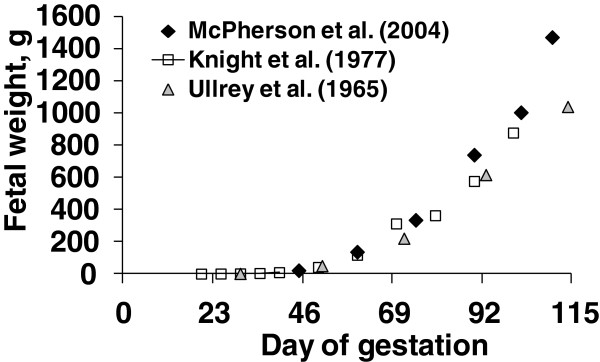
**Growth patterns of porcine fetus.** Adapted from [2,6,7].

Productivity of sows has been changed dramatically during the last decades. Continuous genetic selection led to high prolificacy of sows and production of high lean progeny. As consequences, modern sows produce larger litters than before [1] and each of offspring is leaner and grows faster [2]. A sow currently gives birth to 10 to 16 piglets at birth producing 25 to 30 pigs per year as a litter size has been increased by 3 pigs during 40 years [3]. Recent comparison shows that a porcine fetus is 40% heavier than 40 years ago (Figure 1). However, selection of pigs for high leanness also resulted in high lean type sows possessing a low appetite [4,5].

**Figure 2:**
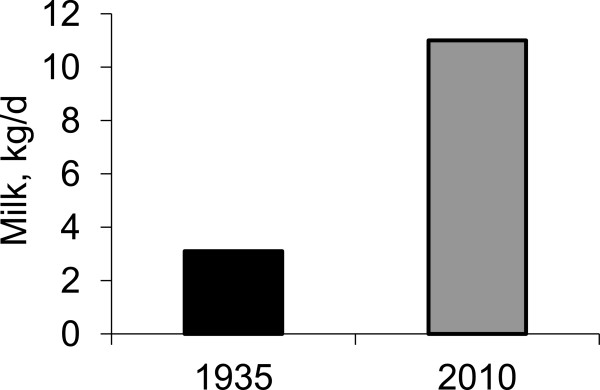
**Milk yield of sows in 1935 and 2010.** Adapted from [8,9].

A sow, therefore, need to produce an ample amount of milk to meet the demands by her large and fast growing litter. In fact, between 1935 and 2010, milk yield has been increased by 4 folds (Figure 2). This suggests that the porcine mammary gland has adapted to support the increased demand for milk production as well. All these improvement with a sow and her litter warrants continuous updates on the nutritional management program. Without proper nutritional supports, sows will face severe catabolic condition. Severe maternal catabolic condition impairs the growth and survival of the litter.

There has been research focused to evaluate the nutrient requirement to meet the accurate needs for the growth of fetus, mammary gland and milk production. This will review how feeding strategies can be adjusted according to the nutrient needs to enhance productivity and health of a sow. Most research data used in this review are based on the studies conducted by the authors between 1996 and 2013.

### Current challenges

**Figure 3:**
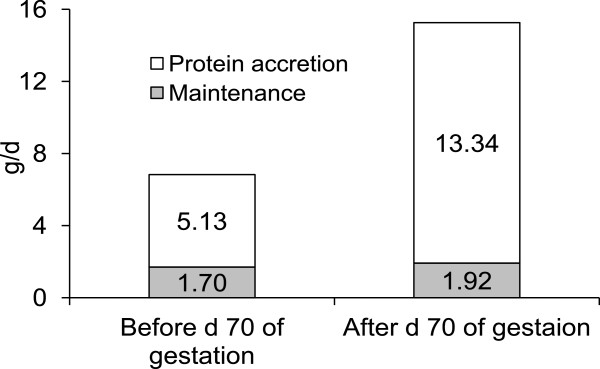
**Requirements of true ileal digestible Lys of sows during early (d 0 to 70 of gestation) and late gestation (from d 70 of gestation to farrowing).** Adapted from [10].

**Figure 4:**
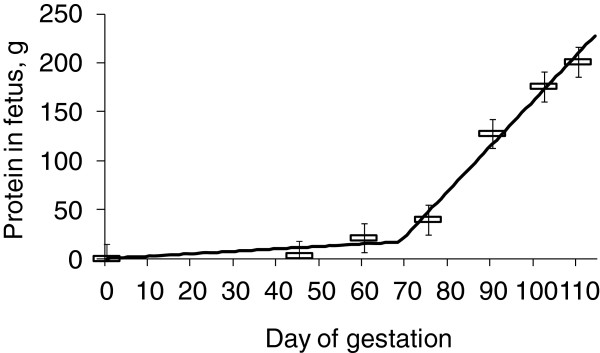
**Protein content in a fetus during gestation.** 0.25 g CP gain per day until d 70 of gestation and 4.63 g CP gain per day from d 70 of gestation. Adapted from [2].

**Figure 5:**
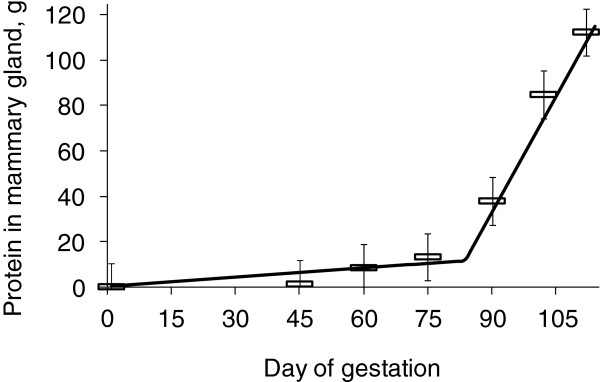
**Protein content in a mammary gland during gestation.** 0.14 g CP gain per day until d 80 of gestation and 3.41 g CP gain per day from d 70 of gestation Adapted from [11].

Conventional feeding program for gestating sows does not provide sufficient proteins and minerals during late gestation causing catabolic condition to sows. Typical corn soybean meal based diets are formulated to provide 8 to 11 g true ileal digestible (TID) Lys daily to sows during the entire gestational period. A recent study [10] shows that conventional feeding program would significantly underfeed Lys during late gestation as requirements of TID Lys increase from 6.8 g/d to 15.3 g/d during late gestation (Figure 3). This increase in Lys requirement is due to dramatic changes in fetal tissue gain from 0.25 to 4.63 g CP/d/fetus [2] and mammary tissue gain from 0.41 to 3.41 g CP/d/gland [11] from early to late gestation (Figures 4 and 5).

**Figure 6:**
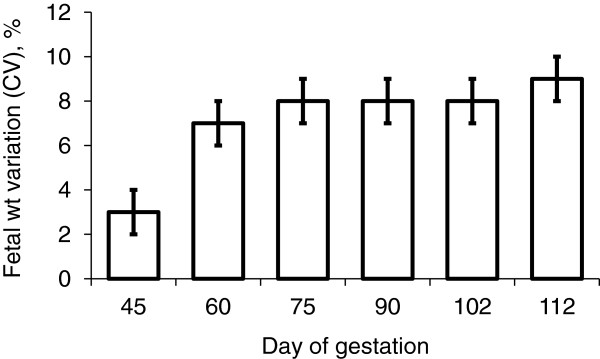
**Weight variations among fetuses within a litter on different days of gestation.** Litter weight variation was expressed as coefficient of variation [CV (%)] for each litter on different days of gestation. Adapted from [10].

**Figure 7:**
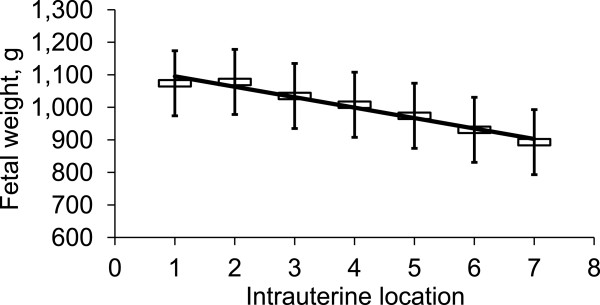
**Litter weight variations among fetuses on d 102 of gestation.** Intra-uterine location 1 indicates cranial (i.e., toward the utero-tubal junction) extremity and 7 indicates cervical (i.e., toward the cervix) extremity. Fetal weight linearly decreased as intra-uterine location changed from 1 to 7: Y = −32.1 x + 1,127.3, R^2^ = 0.97, *P* < 0.001 where Y is fetal weight and x is intra-uterine location (1 to 7). Adapted from [10].

There are several evidences supporting that sows are not providing sufficient nutrients to fetal and mammary growth during late gestation. It has been shown that weight variations expressed as a coefficient of variation (%) among the weights of fetuses in each litter were smaller on d 45 of gestation than those on later than d 60 of gestation [10]. This indicates that fetal growth retardation occurs mainly from d 60 of gestation (Figure 6). Interestingly, fetal weight linearly decreased depending on their location on uterine horn (heaviest toward the utero-tubal junction and the lightest toward the cervix) on d 102 and 112 of gestation whereas there were no correlations between fetal weight and fetal location on d 30 and 60 of gestation (Figure 7). Limited nutrient supply from sows to support the growth of fetuses increased fetal weight variation during late gestation suggesting that current feeding programs for gestating sows is suboptimal for fetal growth especially during late gestation.

Limited nutrient supply to fetus is also contributed from limited blood flow through placenta. Fetal weight variation could be reduced when blood flow was enhanced by dietary supplementation of arginine which can contribute nitric oxide in endothelial cells lining the blood vessels causing vasodilation [12-15].

### Oxidative stress of sows

Catabolic condition increases the production of reactive oxygen species (ROS) causing increased oxidative stress [16,17]. Oxidative damage is a strong indicator of health status and wellbeing of animals in relation to aging, stress, nutritional status, and disease. Increased oxidative stress is responsible for impaired milk production, reproductive performance, and finally longevity of sows [18-20]. Impaired ability to produce milk directly affects the health and growth of nursing piglets, and may also have a long-term effect on health and growth throughout pigs’ life.

**Figure 8:**
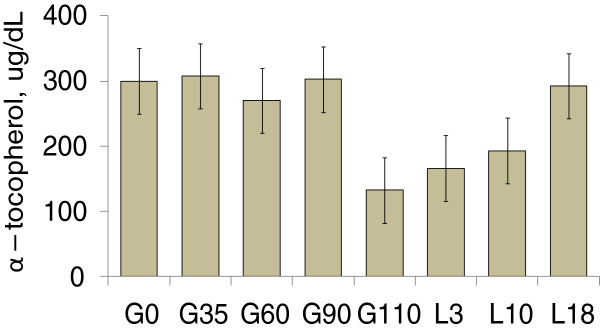
**Plasma α-tocopherol concentration during gestation and lactation.** Adapted from [17].

Recently, it was demonstrated that sows are under severe catabolic status during late gestation causing increased oxidative stress [17]. Plasma α-tocopherol and retinoid concentrations were lower by 56% and 57% at d 110 of pregnancy as compared with d 30 of pregnancy, respectively (Figure 8).

**Figure 9:**
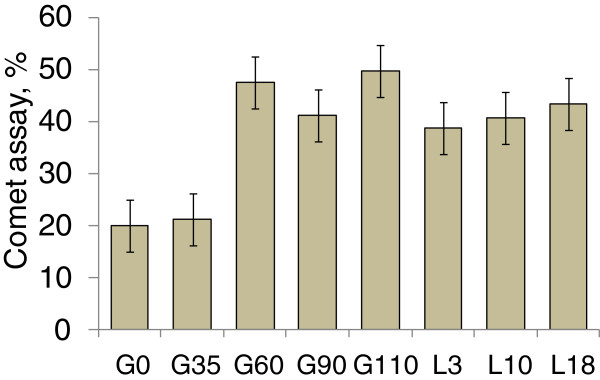
**Lymphocyte endogenous DNA damage (comet assay) during gestation and lactation.** Adapted from [17].

Lymphocyte DNA damage was analyzed by alkaline single cell gel electrophoresis (comet assay) showing increased endogenous DNA damage during late gestation by 125% compared with d 30 of pregnancy (Figure 9). These results indicate that sows undergo increased damage to immune cells due to increased oxidative stress to DNA and also undergo increased systemic oxidative damage due to reduced antioxidative capacity. It seems that oxidative stress to sows increases when sows are under environmental stress such as heat stress and social stress. Sows under heat stress environment showed increased oxidative stress by increased lipid peroxidation, protein oxidation, and oxidative DNA damage compared with the sows under comfort thermal neutral zone. Sows in a gestation stall may have increased oxidative stress compared with sows in a group pen environment. Increased oxidative damages in sows during late pregnancy may negatively affect the growth and health of fetuses as well as postpartum growth of piglets [20].

Hot and humid summer climate causes heat stress reducing reproductive performance and longevity of sows [21,22]. Studies showed that heat stress diminished the secretion of FSH and LH, and delayed puberty in gilts [18,21]. Heat stress may affect early development of embryos [23] causing small litter size, increased number of stillborn, and reduced birth weights [22,24,25]. Studies have shown that lactating sows exposed to high temperatures had reduced feed intake and milk production [26,27]. Hyperthermia from heat stress stimulates reactive oxygen species production causing oxidative damages [28]. A study was recently conducted to investigate the effects of hyperthermal conditions on oxidative status, and reproductive performance of sows during gestation and lactation [19]. A group of sows was under moderate ambient temperature environment (CON) and the others were under high ambient temperature environment (HT).

Sows in HT had decreased (*P* < 0.05) number of piglets born alive and piglets per litter on d 18 of lactation. Litter weight at birth in HT tended to be smaller (*P* = 0.050) compared with those in CON. Litter weight on d 18 of lactation and litter weight gain in HT were smaller (*P* < 0.05) than those in CON. The protein carbonyl concentration in HT was greater than CON on d 90 and 109 of gestation, and d1 and 18 of lactation. Sows in HT had a greater concentration of malonedialdehyde (MDA) on d 90 and 109 of gestation, and d 1 of lactation than sows in CON.

These data indicate that sows in HT have increased lipid and protein damage during late gestation and lactation compared with sows in CON. If comparing oxidative markers between different gestating and lactating days within each treatment, this study showed that sows under heat stress had greater plasma concentrations of 8-OHdG and protein carbonyl on d 109 of gestation than the other days, which indicating that sows under heat stress environment have increased DNA and protein damage during late gestation. Litter weight gain and litter size were negatively correlated (*P* < 0.05) with oxidative stress to sows as indicated by increased plasma concentrations of MDA, protein carbonyls, and 8-OHdG.

Decreased antioxidant capacity during late gestation and lactation can increase oxidative damage by increased production of free radicals when an animal is under high ambient temperature environment [28,29]. This indicates that oxidative stress is one of major stress responses caused by heat stress. This could lead to increased protein turnover due to increased oxidative damage to cellular proteins, increased cell death due to increased peroxidation of membrane lipids and increased DNA mutation and breakdown which can together interfere fetal development, mammary gland development, and milk production as shown as reduced number of piglets born alive, and reduced litter weight gain [19]. Increased oxidative stress during the period of embryonic implantation may cause increased embryonic death which can be related to a reduced litter size for sows under heat stress [19]. Increased oxidative damage to lipid, protein, and DNA was one of the major contributing factors for reduced reproductive performance of sows under a heat stress environment [19].

Gestation crate has widely been used in order to control individual energy intake. However, concerns about animal wellbeing enforce the removal of gestation crates. However, group housing of sows under controlled feed allowance could also potentially increase aggressive behavior between sows, health risks of low dominance sows, occurrence of stereotypic behaviors, and possible reduction in reproductive performance. It has been shown that social and behavioral stresses are associated with physical markers for oxidative stress.

A recent study [20] determined if reproductive performance and oxidative stress status of sows would be affected by different gestational housing systems. Sows were housed either in groups of 3 per pen (PEN) or individual gestational crates (CON) on d 35 of gestation. Reproductive performance of sows housed in gestational pens tended to be inferior to sows housed in gestational crates as indicated by total piglets born per litter and litter weight at born. However, oxidative stress status was not affected by gestational housing indicating that the effects of gestational housing on reproductive performance of sows may not be directly related to oxidative stress status. Oxidative damages to protein and DNA were further increased during late gestation and lactation regardless of gestational housing.

The effect of social rank of gestating sows house in group on their oxidative stress status, immune status, and reproductive performance was also investigated. The social rank of sows within a pen was determined by observing their aggressive behavior for a 4-d period after mixing. Sows within a pen were classified into high-, middle-, and low-ranking groups (HR, MR, and LR) according to their percentage of winning interactions. Sows in LR showed greater (*P* < 0.05) litter size and litter weight than sows in HR even though their BW was inferior (*P* < 0.05) to sows in HR. However, sows in LR has decreased (*P* < 0.05) farrowing rate and increased mortality. Sows in LR had higher (*P* < 0.05) DNA damage compared with HR during late gestation and lactation, which could be a major reason to their poor farrowing rate and increased mortality. The study concluded that the reproductive performance was related to oxidative status of sows regardless which rank they were in.

### Milk production and porcine mammary gland

Strategy to improve milk production should consider enhancing mammary gland growth during gestation and lactation as milk synthesis occurs in a mammary epithelial cell and the number of mammary epithelial cells determines milk production [30]. Nutritional status as well as various factors affect mammary gland growth and therefore milk production. Nutritional management of gestating and lactating sows should consider increased protein and amino acid needs during late gestation and during lactation. Age of sows, litter size, and health status should also be considered in determining nutrient needs for mammary gland growth and milk production.

The growth mammary gland is affected by the anatomical location on a sow. Mammary glands in middle part of the body (typically 4th and 5th pairs of mammary glands) grow faster during gestation and bigger in size at farrowing compared with mammary glands in anterior (1st, 2nd, and 3rd pairs) and posterior (6th, 7th, and 8th pairs) location on a sow [11]. This may be because there is more physical space for mammary glands to grow in middle part of the body whereas anterior and posterior locations which are hindered by legs. It is also speculated that blood supply starts from middle location which is typically 3rd mammary glands and then extends to front and back of the body and thus mammary glands in middle location have greater chance to obtain nutrients compared with those in other locations.

**Figure 10:**
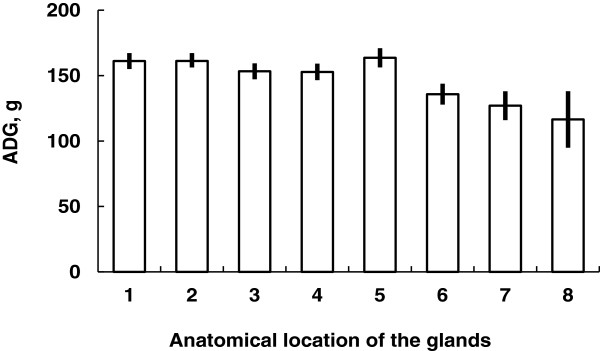
**Growth of piglets depending on anatomical locations of suckling mammary glands [**[31]**].** Average daily gain (ADG) of piglets suckling first 5 pairs of mammary glands was greater (*P* < 0.05) than that of others.

During lactation, however, anterior mammary glands grew faster than others [31]. This may be because anterior mammary glands have a greater preference by piglets during lactation. Growth of suckling piglets was greater when they suckled the first 5 pairs of mammary glands compared with posterior mammary glands (Figure 10). Posterior mammary glands had greater variation in their sizes and milk production whereas anterior and middle mammary glands were more uniform in size and milk production [31].

An increase in litter size directly increase the number of functional mammary glands and thus a sow needs to have increased nutrient supply not just to produce more milk but also to support the growth of these mammary glands that are additionally needed to support the increased litter. Milk yield is more than 50% greater when litter size increased from 6 to 12 [32]. This may result from an increased number of active mammary glands, a crucial component of milk production [33,34]. Sows need an additional 1.0 g lysine per day to account for mammary gland growth for each pig added to a litter [35].

[36] demonstrated that increase in litter size causes decrease in the size of individual suckled mammary glands indicating potential decrease in milk production from each mammary gland. However, mammary glands of a sow with a large litter may be more efficient at producing milk because the reduction in individual pig weight gain was only 73% of the decline in mammary gland growth rate observed in response to increased litter size [36]. Recently, [37] demonstrated that milk lactose content linearly increases, whereas protein content tended to linearly increases when a litter size increased from 8 to 14 piglets indicating changes in litter size affect both milk yield and quality.

Mammary tissue growth continues in suckled glands during lactation in sows [38]. Wet weight of individual suckled mammary gland increases by 55% and total gland DNA doubles during 4 week of lactation. This growth requires 6 g/d true ileal digestible lysine as there is 1 g/d lysine increase in mammary tissue and 5 g/d lysine used for maintenance of mammary tissue [35,39]. Interestingly, the amount of amino acids taken up by the mammary gland does not change with advancing stage of lactation [39] although the number of mammary epithelial cells increases [35] with advancing stages of lactation, indicating a diminished rate of amino acids transport by these cells.

The growth of suckled mammary glands is affected by maternal nutrition. It was shown that mammary growth is affected by both protein and energy intake and it was maximized when sows are provided with 55 g true ileal digestible lysine and 16.9 Mcal metabolizable energy daily during lactation [38]. When considering nutrient recommendation of swine by National Research Council (NRC, 1998), a sow requires 49 g/d lysine. Comparing with [35,36], the difference of 6 g/d lysine (49 vs. 55 g/d) could be accounted for by the need of mammary tissue growth during lactation which was not taken into consideration when NRC recommendation was established in 1998.

### Nutritional management of sows

During gestation, sows undergo dramatic changes with fetal growth and mammary gland growth [2] investigated growth of porcine fetuses and determined their nutrient needs. Growth of fetuses was fairly limited until d 70 of gestation (0.25 g protein increase/d) whereas it was significantly increased (19 folds) to 4.63 g protein increase/d after d 70 of gestation [2,10]. This dramatic increase in growth during late gestation includes growth of heart, liver, intestines, and placenta [2]. Growth of fetuses reported from [2] is about 40% greater than previous report [6,7] indicating improvement of growth potential of fetuses during the last 40 years [11] investigated growth of porcine mammary glands during gestation. It was interesting to observe that growth was not significant until d 80 of gestation (0.41 g protein increase/gland/d) whereas it was significantly increased (24 folds) to 3.41 g protein increase/gland/d after d 80 of gestation [10,11]. Considering this growth patterns, protein requirements should be greater in late gestation compared with early gestation.

In the case of primiparous sows used in this research, it can be estimated that requirements of true ileal digestible (TID) Lys are 6.83 g/d until d 70 of gestation and 15.26 g/d from d 70 of gestation with 2.2 fold difference in the amount [10,40]. Difference in Lys requirement, as an example, is due to dramatic differences in protein gain of fetuses and mammary glands depending on state of gestation. When considering ideal protein concept, Leu and Arg have increased importance during late gestation whereas Thr has increased importance during early gestation [41]. Different growth patterns of fetuses and mammary glands with their unique amino acid compositions contributed to changes in ideal amino acid patterns (Table 1).

**Table 1:** Requirements of true ileal digestible amino acid and ideal dietary amino acid ratios for sows during gestation

	**Amino acid, true ileal digestible**							
	**Lys**	**Thr**	**Val**	**Leu**	**Ile**	**Phe**	**Arg**	**His**
D 0 to 60 of gestation								
Amount, g/d	5.57	4.42	3.62	4.92	3.26	2.79	4.97	2.00
Ratio relative to Lys,%	100.0	79.4	65.0	88.3	58.6	50.1	89.3	35.9
D 60 to 114 of gestation								
Amount, g/d	8.78	6.25	5.83	8.36	4.87	4.54	8.59	3.12
Ratio relative to Lys,%	100.0	71.2	66.4	95.3	55.5	51.8	97.9	35.5

Adapted from [10].

Differences in nutrient requirements based on stages of gestation can create difficulties in feeding practice as it is not feasible to provide two diets if a gestation barn has only one feed line. Top dressing during late gestation can be an alternative way of providing needed nutrients during late gestation [42]. One concern with application of phase feeding during gestation is not to change diets from low to high protein concentration in one day which can cause metabolic stress to pregnant sows. It would be helpful to gradually increase protein or change diets during several days.

During lactation, most sows are under severe catabolic conditions due to produce massive amount of milk with limited nutrient intake [43]. It can be estimated that sows produce 60 g milk/kg body weight which is even greater than dairy cow (50 g milk/kg body weight). Extended catabolic condition during lactation can be a cause of increased oxidative stress [17] negatively affecting longevity and productivity of sows. If voluntary feed intake is a limiting factor leading to the catabolic condition, providing a diet with highly utilizable nutrients would be important.

In swine, the number of mammary epithelial cells is highly correlated to milk production [31]. A study showed that porcine mammary gland continue growing after farrowing and the number of mammary epithelial cells can almost doubled by 3 to 4 week of lactation [35]. Lactating mammary glands have high maintenance requirements for branched chain amino acids [44]. Sows use significant amount of nutrients to support the growth of mammary glands during lactation. As an example, it was calculated that 6 g TID Lys/d is needed to support the growth of mammary gland [35]. A follow-up study further demonstrated that growth of lactating mammary glands was maximized when a primiparous sow consumed 55 g TID Lys and 17 Mcal ME/d [36] which is greater than nutrient requirements by NRC (1998) indicating that it is need to consider the amount of nutrients needed for mammary gland growth when feeding lactating sows.

Young sows with small voluntary feed intake require different quality of proteins compared with old sows with good voluntary feed intake. This is because proteins mobilized from maternal tissues has different amino acid profiles from dietary protein and thus contribute to amino acid balance for milk synthesis. The data show that ideal amino acid pattern, therefore, is different depending on parity of sows (Table 2). Sows with significant loss of body protein need more dietary Thr whereas sows without body protein loss need more dietary Val relative to Lys [10].

**Table 2:** Ideal amino acid patterns and the order of limiting amino acids for lactating sows

Estimated 21-d weight loss (kg)	75 to 80	33 to 45	12 to 15	6 to 8	0	7 to 0
Level of tissue mobilization (%)	50	40	20	5	0	NRC (1998)
Ideal AA pattern (% of Lys)						
Lys	100	100	100	100	100	100
Thr	75	69	63	60	59	62
Val	78	78	78	77	77	85
Leu	128	123	118	115	115	114
Ile	60	59	59	59	59	56
Arg	22	38	59	69	72	56
Order of limiting amino acids						
First	Thr	Lys	Lys	Lys	Lys	Lys
Second	Lys	Thr	Thr	Val	Val	Val
Third	Val	Val	Val	Thr	Thr	Thr

Adapted from [10].

Similar to what it was discussed in feeding gestating sows, differences in nutrient requirements based on parity of sows can create difficulties in feeding practice as it is not feasible to provide multiple diets if a farrowing barn has only one feed line. Top dressing to primiparous sows during lactation can be an alternative way of balancing needed nutrients of sows with different parities. Or grouping sows based on their parity can be another practical way of feeding strategy if a farm has enough number of sows to do parity feeding.

Feed quality seems to be another important factor affecting the productivity of sows. Mycotoxin can cause reduction in voluntary feed intake, systemic inflammation, and increased oxidative stress even at low to moderate level contamination [45,46]. The role of functional nutrients or feed additives was further investigated for their effects on reproductive performance and health status of sows suggesting beneficial effects of selected functional nutrients. Recent publications suggest that these beneficial nutrients can include (1) functional amino acids such as Arg by enhancing blood flow and fetal growth [14,15,47] and Trp by reducing oxidative and behavioral stress [48,49], (2) omega-3-fatty acids by reducing systemic inflammation [50,51], and (3) yeast cell contents by enhancing milk production [9,42,52].

## Conclusion

As discusses so far, nutrient requirements of sows are affected by stage of gestation and parity of sows. Dietary antioxidant concentrations need to be re-evaluated for its sufficiency in sow diets especially to prevent excessive oxidative stress during late gestation and lactation. When feeding sows, consideration of phase feeding of gestating sows and parity feeding of lactating sows could enhances production longevity and health of sows. Use of selected nutrients and additives seems to help productivity and health of sows.

## Competing interests

The authors declare that they have no competing interests.

## Authors’ contributions

SWK, ACW, YBS, and YZ contributed to this review paper by collecting data and writing the text. All authors read and approved the final manuscript.
